# Mild exacerbation of obesity- and age-dependent liver disease progression by senolytic cocktail dasatinib + quercetin

**DOI:** 10.1186/s12964-021-00731-0

**Published:** 2021-04-08

**Authors:** Marco Raffaele, Kristina Kovacovicova, Jan Frohlich, Oriana Lo Re, Sebastiano Giallongo, Jude A. Oben, Martin Faldyna, Lenka Leva, Antonino Giulio Giannone, Daniela Cabibi, Manlio Vinciguerra

**Affiliations:** 1 International Clinical Research Center, St. Anne’s University Hospital, Brno, Czech Republic; 2 Department of Biology, Faculty of Medicine, Masaryk University, Brno, Czech Republic; 3 Institute for Liver and Digestive Health (ILDH), Division of Medicine, University College London (UCL), London, UK; 4 Veterinary Research Institute, Brno, Czech Republic; 5 Department of Health Promotion, Mother and Child Care, Internal Medicine and Medical Specialties, Pathologic Anatomy Unit-University of Palermo, Palermo, Italy; 6 ERA Chair in Translational Stem Cell Biology, Medical University of Varna, Varna, Bulgaria

**Keywords:** Senolytics, Liver diseases, Inflammation, Cancer, Obesity

## Abstract

**Background:**

Nonalcoholic fatty liver disease (NAFLD) is increasingly prevalent and represents a growing challenge in terms of prevention and treatment. A minority of affected patients develops inflammation, subsequently fibrosis, cirrhosis and hepatocellular carcinoma (HCC). HCC is a leading cause of cancer-related death. An increased number of senescent cells correlate with age-related tissue degeneration during NAFLD-induced HCC. Senolytics are promising agents that target selectively senescent cells. Previous studies showed that whereas a combination of the senolytic drugs dasatinib and quercetin (D + Q) reduced NAFLD in mice, D + Q lacked efficacy in removing doxorubicin-induced β-gal-positive senescent cells in human HCC xenografted mice. Whether D + Q has an effect on the age-associated spectrum of NAFLD-inflammation-HCC remains unknown.

**Methods:**

Here, we utilized an established model of age- and obesity-associated HCC, the low dose diethylnitrosamine (DEN)/high fat diet (HFD), a regimen promoting liver inflammation and tumorigenesis over a long period of 9 months. Four groups of mice each were created: group 1 included control untreated mice; group 2 included mice treated with D + Q; group 3 included mice undergoing the DEN/HFD protocol; group 4 included mice undergoing the DEN/HFD protocol with the administration of D + Q. At the end of the chemical/dietary regimen, we analyzed liver damage and cell senescence by histopathology, qPCR and immunoblotting approaches.

**Results:**

Unexpectedly, D + Q worsened liver disease progression in the DEN/HFD mouse model, slightly increasing histological damage and tumorigenesis, while having no effect on senescent cells removal.

**Conclusions:**

In summary, using an animal model that fully recapitulates NAFLD, we demonstrate that these compounds are ineffective against age-associated NAFLD-induced HCC.

**Video Abstract**

**Supplementary Information:**

The online version contains supplementary material available at 10.1186/s12964-021-00731-0.

## Background

The majority chronic diseases appear with increasing age and thus have high prevalence in the elderly [1, 2]. Similarly, geriatric conditions, such as mild cognitive impairment and frailty drastically augment with the aging of the individual [3]. Non-alcoholic fatty liver disease (NAFLD) is the most common chronic liver disorder, and it affects > 25% of the general population [4]. NAFLD is the main risk factor to develop fibrosis, cirrhosis and hepatocellular carcinoma (HCC), a devastating disease [5]. The incidence of NAFLD increases with age [5]. A great bulk of evidence shows that the progressive accumulation of senescent cells can mark and drive age-associated alterations and pathologies [6–8]. Senescence is a cellular response characterized by a stable growth arrest and other phenotypic alterations that include upregulation of tumor suppressor p16 and a proinflammatory secretome (senescence-associated secretory phenotype, SASP) [9]. Senescence plays roles in normal development, maintains tissue homeostasis, and limits tumor progression. Rapid gain of interest in cellular senescence is rising from the potential of therapeutically targeting this process, in order to improve age-related pathologies, using drugs called senolytics [10–13]. These agents include dasatinib (D), quercetin (Q), fisetin, Navitoclax (ABT-263) and others [10–12]. Senescence associated β-galactosidase (SA-β-gal) – the most common marker of cellular senescence [14]—assays in human or murine fibroblasts are increasingly used as high-throughput screening platforms to rapidly identify new senolytics [15, 16], for further testing. Nonetheless, the first and best-studied senolytic drugs, D and Q, were discovered using a mechanism-based approach instead a high-throughput screening [17]. It was shown that cellular senescence drives NAFLD and the mix Dasatinib (5 mg/kg) + Quercetin (50 mg/kg) dependent elimination of p16 overexpressing senescent cells may be a novel therapeutic strategy to reduce NAFLD and obesity-induced anxiety in mice models. The same authors reported that "hit-and-run" treatment with D + Q, which have elimination half-lives < 11 h, significantly decreases senescent cell burden in humans, in particular in p16 highly expressing cells (p16^high^) in the adipose tissue. On the contrary, we demonstrated that D + Q used at the same concentration of these previous studies [17–21], are ineffective, alone or in synergy with senescence-inducing chemotherapy, in mice xenografted with human HCC cells [22]. However, HCC xenografts present several approximations, including immuno-deficient hosts, non-natural tumor site (sub-cutaneous) and lack of genetic diversity. Moreover, they do not feature the progression of liver diseases. The current study was designed to assess the role of D + Q in a comprehensive and robust mouse model of obesity- and age- dependent liver disease progression, developing NAFLD and HCC [23, 24].

## Results

### Dasatinib + Quercetin (D + Q) worsens liver disease progression in the diethylnitrosamine (DEN) / high fat diet (HFD) mouse model

**Fig. 1:**
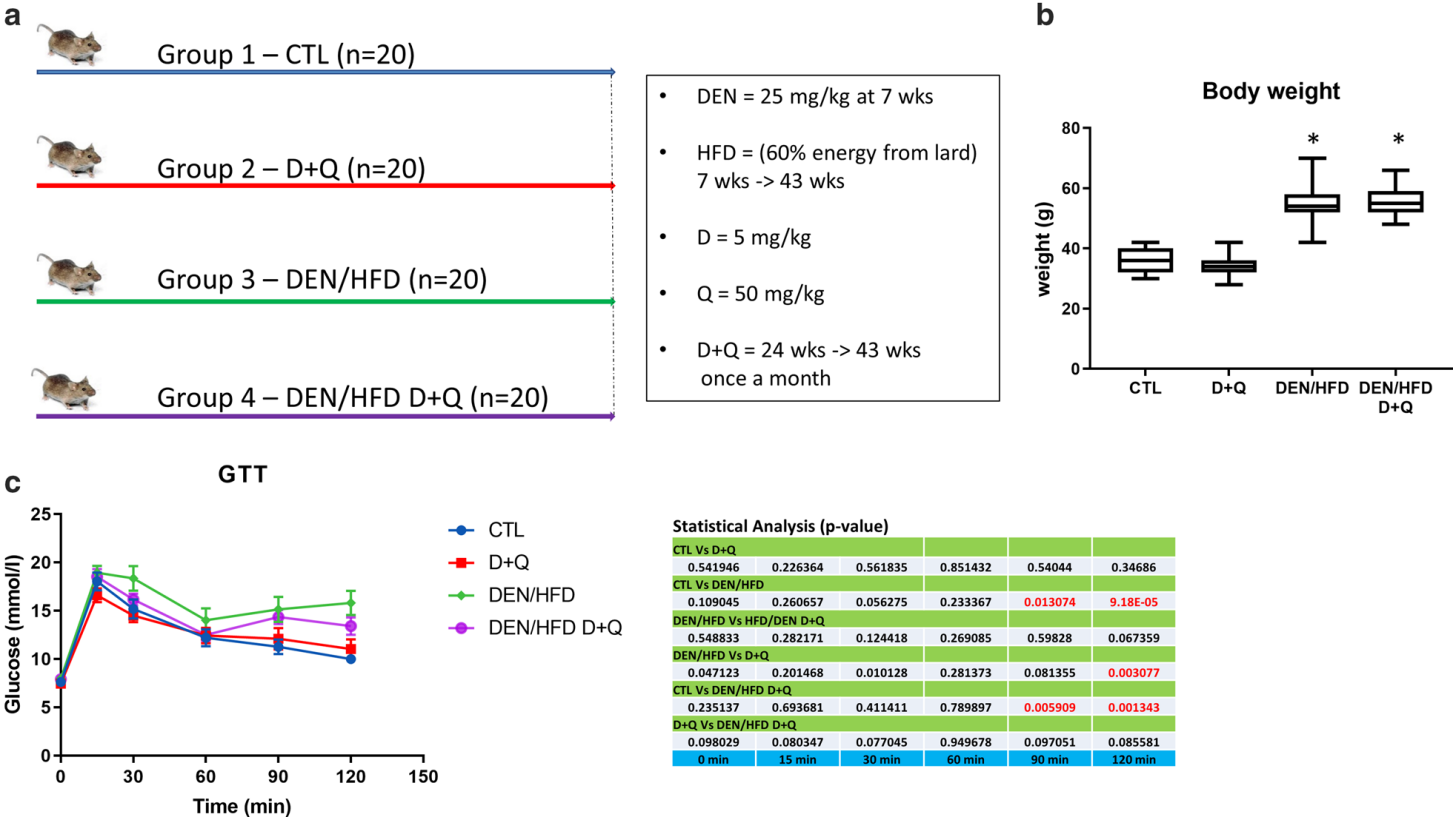
Effects of D + Q treatment on diethylnitrosamine (DEN) / high fat diet (HFD) mouse model. **a** Experimental design of the study. **b** Body weight of mice under control diet or DEN/HFD and treated with D + Q (C) Intraperitoneal glucose tolerance test (GTT) graph and *p* value table showing the statistical significance among all the groups. *p < 0.05 Vs CTL determined by Student´s t test

**Fig. 2:**
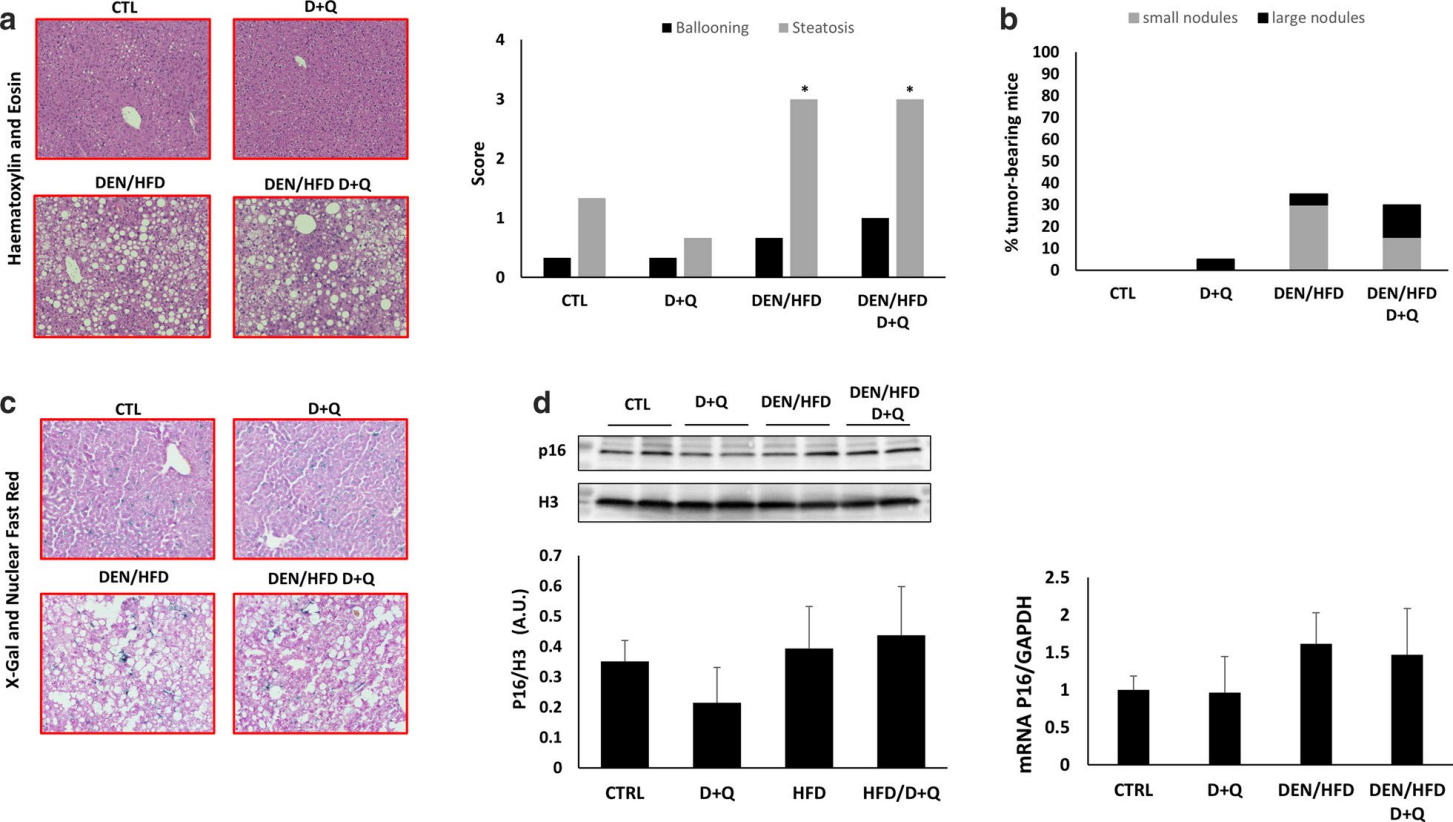
Effects of D + Q treatment worsen liver disease progression. **a** Representative liver H&E images from control diet and DEN/HFD mice treated with D + Q and steatosis/ballooning score graph. **b** Distribution of tumor-bearing mice among the groups, with classification of dysplastic nodules based on the size in small nodules (diameter < 3 mm) and large nodules (diameter > 3 mm). **c** Representative liver β-gal staining + nuclear fast red (NFR) images of CTL, D + Q, DEN/HFD and HFD/DEN D + Q groups showing no difference in senescent positive cells. **d** Immunoblotting and qRT-PCR analysis of the senescence marker p16. *p < 0.05 Vs CTL determined by Student´s t test

**Fig. 3:**
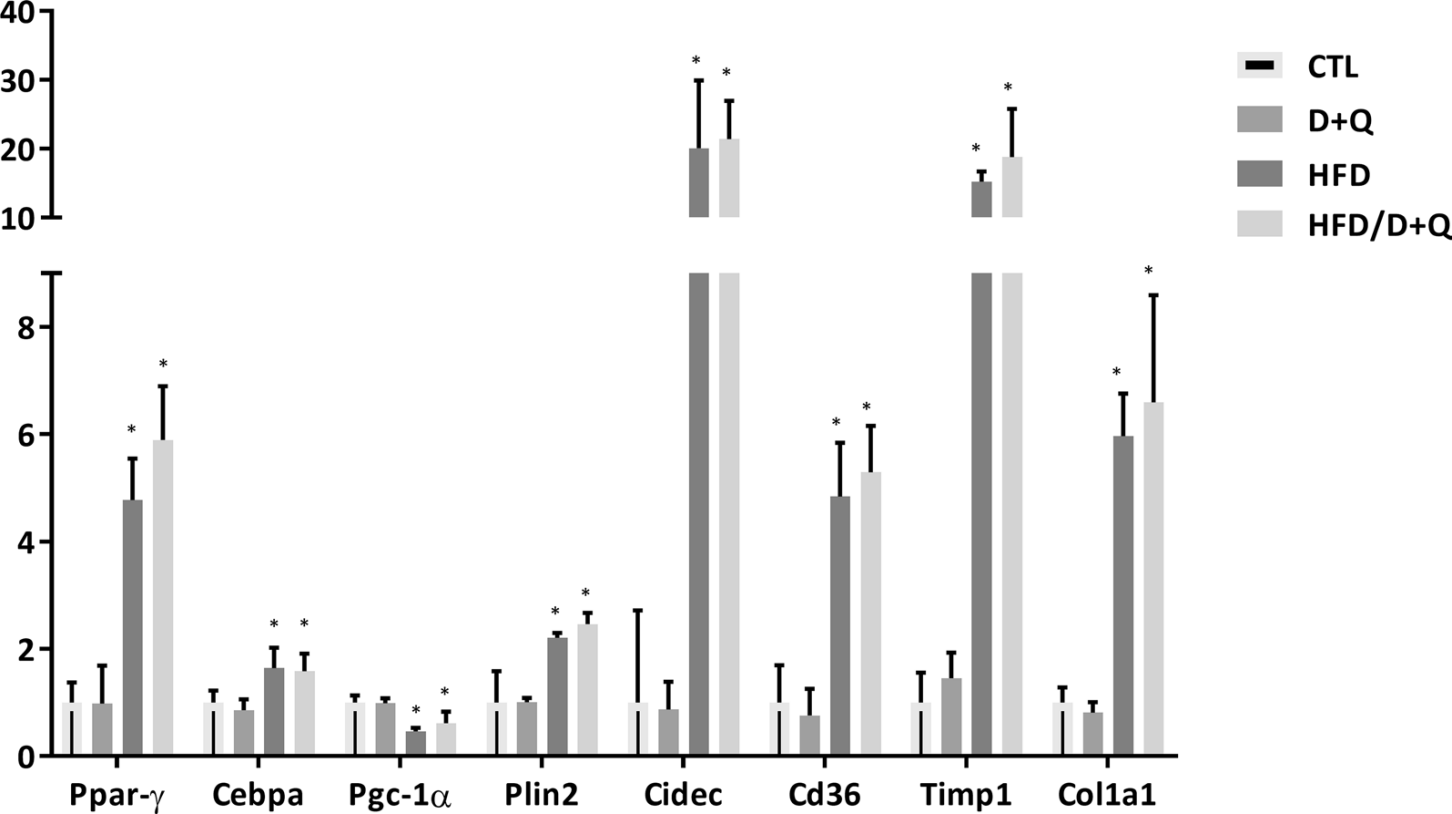
D + Q does not affect NAFLD key factors gene expression in liver of both lean and obese mice. mRNA expression of Ppar-γ, Cebpa, Pgc-1α, Plin2, Cidec, Cd36, Timp1 and Col1a1 of control mice, D + Q treated group, DEN/HFD group and DEN/HFD mice treated with D + Q. *p < 0.05 Vs CTL determined by Student’s t test

Cellular senescence was shown to drive NAFLD and D + Q treatment could alleviate this pathology [18–20]. However, these previous reports did not analyze the effectiveness of these senolytics in hampering the progression of NAFLD into inflammatory states and, ultimately, into HCC. To test this hypothesis, we adopted a well-established mouse model of NAFLD-induced HCC, where young mice (7 weeks of age) are administered with a low dose of a carcinogen (25 mg/kg diethynitrosamine, DEN), followed by feeding a high fat diet (HFD, 60% energy from lard)) from the 7th week to the 43rd week, when the animals were sacrificed for further analyses [23, 24] (Fig. 1a). Four groups of 20 mice each were created: group 1 included control untreated mice; group 2 included mice treated with D + Q from the 24th week to the 43rd week of age (D = 5 mg/kg; Q = 50 mg/kg); group 3 included mice undergoing the above mentioned DEN/HFD protocol; group 4 included mice undergoing the DEN/HFD protocol with the administration of D + Q (Fig. 1a). At the end of the chemical/dietary regimen, the DEN/HFD groups 3 and 4 exhibited a ~ 50% increase in body weight, with not tangible effect of D + Q treatment (Fig. 1b). Blood analysis showed no differences in number of white blood cell and red blood cell count as well as in hemoglobin levels, among the four groups (Table 1). Upon HFD feeding, biochemical plasma analyses detected a significant increase in the levels of LDL, HDL, TC, ALT and AST, while we observed a paradoxical decrease in TG – most likely due to a reduced dietary carbohydrate [25], compared to control diet (Table 2). None of these changes were affected by D + Q treatment (Table 2). We then investigated the ability of the four groups of mice to respond to a glucose challenge, as readout of metabolic health. In glucose tolerance tests (GTT), glucose levels were significantly higher in DEN/HFD mice (group 3) at 90 and 120 min time points, compared to control littermates (group 1); notably, D + Q administration (in groups 2 and 4) did not have any significant effect on GTT curves (Fig. 1c). We then conducted histopathological assessment of D + Q effects on liver parenchymal architecture. Figure 2a shows representative images of Hematoxylin & Eosin (H&E) stained liver sections of mice fed with control diet or HFD and treated with D + Q. Steatosis reached the maximum score of 3 in the HFD, while a value of 1 was assigned to the control diet groups identifying a mild steatosis probably caused by aging. Several hepatocyte ballooning was also noticed in the livers of HFD mice, while only a small number was detected in liver control-diet mice livers. In both diet groups D + Q did not show to improve liver health, obtaining similar steatosis and ballooning scores compared to their respective controls. The increased fat storage in the liver of mice fed with HFD was corroborated by the increased expression levels of key lipogenic genes Ppar-γ and Cebpa, the lipid droplets growth related genes Plin2, Ceidec and the main membrane fatty acid transporter Cd36; while Pgc-1α, a master regulator of the mitochondrial functions was downregulated in the groups upon HFD feeding, compared to control (Fig. 3) [26]. In accordance with the fibrotic processes that characterized the progression of NADLF, the levels of the markers Col1a1 and Timp1 were strongly upregulated in the HFD groups compared to control [27]. None of these changes were affected by D + Q treatment (Fig. 3). Dysplastic nodules were observed in many mice liver and classified based on their size, in small nodules (< 3 mm) and large nodules (> 3 mm) (Fig. 2b). About 35% of HFD mice livers pre-treated with DEN and, surprisingly, 5% of lean mice treated with D + Q, were presenting nodules, while none was detected in the lean controls. Interestingly, whereas the percentage of tumor-bearing mice in HFD/DEN control and treated group was similar, a difference in small and large nodules ratio was noted, with an increased presence of large nodules in the HFD/DEN D + Q group. The senolytic effect of D + Q treatment was assessed by SA-β-gal staining (Fig. 2c), and by gene/protein expression measurement of the senescence marker p16 (Fig. 2d). Contrary to previous studies [18], the analysis of B-gal staining images did not reveal any significant difference in senescence positive cells between the treated mice and their control, as shown in the representative images in Fig. 2c. Supporting the SA-β-gal staining, the evaluation of p16 gene and protein levels did not show significant differences among the groups (Fig. 2d). These findings suggest that D + Q is not effective in removing senescent cells from adult mice (11 months) liver, further showing a slight pro-tumorigenic activity in accordance with our previous finding [22].

**Table 1:** Blood count analyses

Group (N = 8)	RBC (10^12^/l)	Hct (%)	Hgb (g/L)	Leukocytes (10^9^/L)	Lymphs (10^9^/L)	Neutrophils (10^9^/L)	Band cells (10^9^/L)	Monocytes (10^9^/L)	Eosinophils (10^9^/L)	Basophils (10^9^/L)
CTL	7.99 ± 1.7	37.21 ± 5.18	124.14 ± 17.67	13.67 ± 2.86	11.92 ± 2.73	1.43 ± 0.49	0.06 ± 0.06	0.001 ± 0.02	0.24 ± 0.17	0.01 ± 0.02
D + Q	7.67 ± 0.53	35.78 ± 3.05	120.29 ± 10.05	11.99 ± 3.76	10.35 ± 4.02	1.74 ± 1.16	0.04 ± 0.05	0.016 ± 0.04	0.30 ± 0.19	0.01 ± 0.02
HFD	7.75 ± 0.61	36.81 ± 2.30	122.88 ± 8.17	15.27 ± 3.96	12.88 ± 3.14	2.07 ± 1.03	0.05 ± 0.06	0.10 ± 0.17	0.16 ± 0.12	0.01 ± 0.03
HFD/D + Q	7.69 ± 1.23	36.2 ± 5.41	120.75 ± 17.75	15.09 ± 5.67	13.17 ± 5.50	1.79 ± 0.82	0.03 ± 0.06	0.02 ± 0.04	0.07 ± 0.1	0.01 ± 0.02

*RBC* red blood cells, *Hct* hematocrit, *Hgb* hemoglobin, *Lymphs* Lymphocytes

**Table 2:** Plasma analyses

Group (N = 6)	LDL (mmol/L)	HDL (mmol/L)	TC (mmol/L)	TG (mmol/L)	ALT (µkat/L)	AST (µkat/L)
CTL	0.40 ± 0.09	2.4 ± 0.41	3.9 ± 0.45	1.16 ± 0.24	1.50 ± 0.49	0.44 ± 0.12
D + Q	0.37 ± 0.06	2.52 ± 0.54	3.64 ± 0.42	0.96 ± 0.22	1.72 ± 0.96	0.66 ± 0.49
HFD	0.63 ± 0.07*	4.56 ± 0.28*	6.70 ± 0.47*	0.88 ± 0.06*	3.09 ± 0.99*	4.33 ± 1.56*
HFD/D + Q	0.65 ± 0.07*	4.66 ± 0.11*	6.98 ± 0.19*	0.80 ± 0.06*	2.98 ± 0.59*	4.47 ± 0.93*

*LDL* low-density lipoprotein, *HDL* high-density lipoprotein, *TC* total cholesterol, *TG* triglycerides, *ALT* alanine transaminase, *AST* aspartate transaminase

^*^p < 0.05 vs CTL Group

## Methods

### Mice models

All experiments were carried out following the rules of reduction of numbers of animals and minimizing their suffering during the experiments.

*D* + *Q treatment:* all animal work was conducted either according to Act No 246/1992 Coll., on the protection of animals against cruelty and was approved by the Central Commission for Animal Welfare, approval ID 2989/2018 (Ministry of Agriculture, Czech Republic). Male C57/BL6J mice were obtained from AnLab (Czech Republic) at the age of 5 weeks and were maintained in a pathogen-free facility under temperature- and light- controlled conditions (22 + 2 °C, 12 h light/dark regimen) with free access to food and water. The animals were fed by control diet (CD.88137; ssniff Spezialdiäten GmbH) or by experimental high-fat diet (60% of kj has the fat origin; D12492; ssniff Spezialdiäten GmbH). Mice were divided into following groups: (1) control group: mice given a placebo treatment; (2) D + Q group: mouse given dasatinib plus quercetin (D + Q) treatment; (3) DEN/HFD group: the initial liver damage was induced by single administration of DEN and animals were fed by high fat diet [23]; (4) DEN/HFD D + Q group: the same liver damage induction as in group 3 plus D + Q treatment. Mice from group 3 and 4 (model of steatosis and steatohepatitis) obtained single i.p. injection of diethylnitrosamine (DEN; 25 mg/kg; Sigma Aldrich) at the age of 7 weeks. The intervention of senolytics or placebo started at the 6 months of age and it was performed once per month over a 5-month period. The solution of dasatinib (5 mg/kg; Sigma Aldrich) and quercetin (50 mg/kg; Sigma Aldrich) or the placebo was administrated by oral gavage in 100–150μL. Glucose tolerance test (GTT) was performed one week before the sacrifice, after 12 h of fasting by i.p. administration of 1.5 g/kg of glucose solution (Sigma Aldrich). Blood glucose levels were obtained at time points 0 (basal level); 15; 30; 45; 60 and 120 min measured by ACCU-CHEK Performa glucometer (Roche). Blood samples were collected during the sacrifice and hematological and biochemical parameters were analyzed by hematological analyzer BC-2800 Vet and biochemical analyzer BS200 (Mindray, Shenzhen, PRC), respectively, according to manufacturer´s recommendations.

### Histology

Samples of livers were harvested from euthanized mice, fixed with 4% paraformaldehyde, processed for the embedding in tissue freezing medium (Leica) or paraffin wax (Surgipath Paraplast Plus, Leica) and sectioned for histopathological analysis. The paraffine liver sections were stained with Hematoxylin–Eosin (H&E; Sigma) for overall assessment of parenchymal architecture, hepatocyte abnormalities (including ballooning), inflammatory infiltration and the measurement of lipid droplets size. These features were scored according to the NAFLD histologic activity score (NAS) system [28]. Briefly, double-blinded analysis identified the score of steatosis (grade 0 ≤ 5%; 1 = 5–33%; grade 2 = 34%–66%; grade 3 ≥ 66%), lobular inflammation (0: no foci, 1 < 2 foci per 200 × field, 2: 2 to 4 foci per 200 × field, and 3: > 4 foci per 200 × field), hepatocyte ballooning (0: none; 1: rare or few; 2: many). Fibrosis was quantified using ImageJ software (NIH) and expressed as % of the total area of the section. All analyses were performed in triplicate by two independent pathologists (A.G.G. and D.C.).

### X-gal staining

B-galactosidase detection method was performed as previously described [14]. Briefly, tissues frozen sections were fixed in 1% formalin in PBS for 1 min at RT, washed three times in PBS and incubated overnight on X-gal staining solution [1 mg/mL of X-gal (VWR), 40 mM citric acid/sodium phosphate buffer, 5 mM potassium ferricyanide (Sigma), 5 mM potassium ferrocyanide (Sigma), 150 mM NaCl, and 2 mM MgCl2] at 37 °C in a humidified chamber. The experiments were carried out using staining solutions at pH 6.0 to assess the SA-β-gal activity. Samples were rinsed with distilled water and counterstained with Nuclear Fast Red (Sigma) for 5 min. Images were acquired using Pia-Apochromat 20 × 0.8 M27 objective on Axio scan Z1 (Zeiss).

### qRT-PCR

RNA extraction and qRT-PCR methods were previously described [29, 30]. Murine primer sequences were listed in Table 3.

**Table 3:** qRT-PCR primers (Mus Musculus)

Gene	Forward (5′–3′)	Reverse (5′–3′)
Gapdh	AGGTCGGTGTGAACGGATTTG	TGTAGACCATGTAGTTGAGGTCA
p16	CGCTCTGGCTTTCGTGAACAT	GCCCATCATCATCACCTGGTC
Ppar-γ	TCGCTGATGCACTGCCTATG	GAGAGGTCCACAGAGCTGATT
Cebpa	ACTCCTCCTTTTCCTACCG	AGGAAGCAGGAATCCTCC
Pgc-1α	TCCCATACACAACCGCAGTC	ACCCTTGGGGTCATTTGGTG
Plin2	GACCTTGTGTCCTCCGCTTAT	CAACCGCAATTTGTGGCTC
Cidec	ATGGACTACGCCATGAAGTCT	CGGTGCTAACACGACAGGG
Cd36	GAGCAACTGGTGGATGGTTT	GCAGAATCAAGGGAGAGCAC
Timp1	GCAACTCGGACCTGGTCATAA	CGGCCCGTGATGAGAAACT
Col1a1	GCTCCTCTTAGGGGCCACT	CCACGTCTCACCATTGGGG

### Immunoblotting analyses

Frozen liver tissues were ground under liquid nitrogen and resuspended in RIPA lysis buffer (ThermoFisher) that included protease and phosphatase inhibitors cocktails (Sigma P5726 and P8340). Immunoblotting analyses were performed as previously described [31–33]. Primary Antibodies were purchased from Abcam (CDKN2A/p16INK4a, ab189034) and Cell Signaling (Histone H3, 9715S). Anti-rabbit IgG HRP-linked (Cell Signaling, 7074S) was used as secondary antibody. Immuno-positive bands were visualized by chemiluminescence (GE Healthcare Life Sciences) using the Molecular Imager ChemiDoc xrs + system (Bio-Rad) and quantified by densitometry analysis performed after normalization with H3. Results were expressed as arbitrary units (AU).

### Statistical analyses

Results are expressed as means ± SD. Comparisons between groups were performed with the parametric Student's t-test, using GraphPad Prism Software (version 5.00 for Windows, San Diego, CA, USA): *p* value ≤ 0.05 was considered significant.

## Discussion

The process of aging predisposes to hepatic functional and structural impairment [5]. The most common liver disease, NAFLD can evolve into NASH in the presence of oxidative stress and inflammation [34, 35]. NASH is a serious risk factor for disabling liver diseases such as cirrhosis and HCC. Old age seems to favor NAFLD, NASH, and ultimately HCC, in agreement with the inflamm-aging theory [5]. Others and we have observed an increase in the amount of senescent hepatocytes and other hepatic parenchymal cells during the transition from NAFLD to HCC [32, 36]. In the context of liver disease progression, the concept of selective elimination of senescent cells using senolytics holds a great therapeutic potential [37]. Our present and past work demonstrates that the first generation and best-characterized senolytic cocktail D + Q, at the same dosage used in literature, is largely ineffective in preventing HCC development and growth in vivo [22]. On the opposite, it is of concern the observation that D + Q administration has slight pro-tumorigenic effects in these same models. These data are in marked contrast with the report that treatment with a combination of D + Q reduces NAFLD in HFD-fed mice [18]. However, the latter study did not analyze liver disease progression beyond NAFLD. Recently, Grosse et al*.* elegantly showed that D + Q combined treatment reduced the number of senescent p16^high^ macrophages in the liver and in the adipose tissue, but it had no effect on liver endothelial sinusoidal cells or on adipocytes [38]. In support of our finding, Gross et al*.* strikingly found that systemic elimination of p16^high^ senescent cells in 10–12 months old mice triggered liver, perivascular tissue fibrosis and general health deterioration [38]. Overall, these controversies suggest that while elimination of some senescent cells is beneficial for healthy aging and overall lifespan [39], it is very important to identify which senescent cells are targeted by specific senolytics and their overall effects on health span.

## Conclusions

In summary, using an animal model that fully recapitulates NAFLD, we demonstrate that D + Q treatment is ineffective against age-associated NAFLD-induced HCC. The clinical use of these compounds awaits safety, target identification, and efficacy studies to determine the optimal dosage required to clear senescent cells.

## Data Availability

All the data generated or analyzed for this project are included in this article.

## Abbreviations

NAFLD: Nonalcoholic fatty liver disease
NASH: Nonalcoholic steatohepatitis
HCC: Hepatocellular carcinoma
D: Dasatinib
Q: Quercetin
DEN: Diethylnitrosamine
HFD: High fat diet
SASP: Senescence-associated secretory phenotype
SA-β-gal: Senescence associated β-galactosidase
GTT: Glucose tolerance test
H&E: Hematoxylin & Eosin
NAS: Histologic activity score
PCR: Polymerase chain reaction
HRP: Horseradish peroxidase
H3: Histone H3
p16: Cyclin Dependent Kinase Inhibitor 2A (CDKN2A/p16INK4a)
Gapdh: Glyceraldehyde 3-phosphate dehydrogenase
Ppar-γ: Peroxisome proliferator-activated receptor gamma
Cebpa: CCAAT enhancer binding protein alpha
Pgc-1α: Peroxisome proliferator-activated receptor gamma coactivator 1-alpha
Plin2: Perilipin-2
Cidec: Cell death inducing DFFA like effector C
Cd36: Cluster of differentiation 36, also known as fatty acid translocase (FAT)
Timp1: Tissue inhibitor of metalloproteinases 1
Col1a1: Collagen type I alpha 1 chain
